# Prevalence of risk factors for foot ulceration in a general haemodialysis population

**DOI:** 10.1186/1757-1146-3-S1-O13

**Published:** 2010-12-20

**Authors:** Nia Jones, Stephen Riley, Aled Phillips

**Affiliations:** 1 Cardiff and Vale University Health Board, Cardiff, UK

## Background and objectives

Lower limb complications contribute to the significantly increased morbidity and mortality associated with renal replacement therapy. This audit was designed to document the prevalence of known risk factors for foot ulceration in a stable haemodialysis population.

## Design, setting, participants and measurements

A dedicated podiatric service was introduced into a satellite haemodialysis unit. All patients were invited to have a session with the podiatrist to have a formal assessment of their feet. The Renal Foot Screening Tool was developed to prospectively identify risk factors associated with foot ulceration present in individual patients. This included identification of peripheral neuropathy, peripheral arterial disease, and structural foot pathology. In addition basic demographic and co-morbidity data was collected.

## Results

Of 57 patients screened 24 had diabetes mellitus. Peripheral neuropathy was identified in 50% and 18% of diabetic and non-diabetic patients respectively. Peripheral arterial disease as documented by Doppler studies was documented in 45% of diabetic and 30% of non-diabetic patients. A high percentage (79%) of patients had some form of structural foot pathology. Forty nine percent of the total cohort had 2 or more risk factors for foot ulceration (16/24 diabetic v 12/33 non-diabetic).

## Conclusions

There is a high prevalence of risk factors for foot ulceration in this group of haemodialysis patients. This suggests these patients are at high risk of future foot ulceration, which may lead to amputation. Further work on strategies to monitor and prevent foot pathology may lead to a reduction in morbidity and mortality associated with foot ulceration.

